# There Is No Evidence for a Temporal Link between Pathogen Arrival and Frog Extinctions in North-Eastern Australia

**DOI:** 10.1371/journal.pone.0052502

**Published:** 2012-12-27

**Authors:** Ben L. Phillips, Robert Puschendorf, Jeremy VanDerWal, Ross A. Alford

**Affiliations:** School of Marine and Tropical Biology, Centre for Tropical Biodiversity and Climate Change, James Cook University, Townsville, Queensland, Australia; Tuscia University, Italy

## Abstract

Pathogen spread can cause population declines and even species extinctions. Nonetheless, in the absence of tailored monitoring schemes, documenting pathogen spread can be difficult. In the case of worldwide amphibian declines the best present understanding is that the chytrid fungus *Batrachochytrium dendrobatidis* (Bd) has recently spread, causing amphibian declines and extinction in the process. However, good evidence demonstrating pathogen arrival followed by amphibian decline is rare, and analysis of putative evidence is often inadequate. Here we attempt to examine the relationship between Bd arrival and amphibian decline across north-eastern Australia, using sites where a wave-like pattern of amphibian decline was first noticed and at which intensive research has since been conducted. We develop an analytical framework that allows rigorous estimation of pathogen arrival date, which can then be used to test for a correlation between the time of pathogen arrival and amphibian decline across sites. Our results show that, with the current dataset, the earliest possible arrival date of Bd in north-eastern Australia is completely unresolved; Bd could have arrived immediately before sampling commenced or may have arrived thousands of years earlier, the present data simply cannot say. The currently available data are thus insufficient to assess the link between timing of pathogen arrival and population decline in this part of the world. This data insufficiency is surprising given that there have been decades of research on chytridiomycosis in Australia and that there is a general belief that the link between Bd arrival and population decline is well resolved in this region. The lack of data on Bd arrival currently acts as a major impediment to determining the role of environmental factors in driving the global amphibian declines, and should be a major focus of future research.

## Introduction

Emerging infectious diseases are increasingly recognised as a serious and growing threat to biodiversity [1], [2]. While there are now many well-documented cases of population collapses and extirpations associated with disease [3]–[6], data linking the arrival of a pathogen and the timing of the host population collapse are relatively rare. This rarity stems from the inherent difficulty of screening for as yet unidentified pathogens, problems in detecting diseases [7], and a lack of established monitoring projects and funding [8]. In particular, when an infectious disease is newly emergent, it often takes time to identify the pathogen and develop tailored monitoring strategies. Thus, many populations can decline or even go extinct before it is possible to effectively monitor for the presence or absence of the causal pathogen.

One of the best-studied globally emergent diseases of conservation concern is amphibian chytridiomycosis. Globally, and in the last thirty years, many species of amphibians have declined in numbers and some have even gone extinct [9]–[12]. Although widespread across the planet, these declines also often exhibited clear spatiotemporal patterns: they were associated with high elevations and some appear to have advanced in a wave-like manner towards the equator [13]–[15]. In 1999 the chytrid fungus *Batrachochytrium dendrobatidis,* (Bd, [16]) was identified as an amphibian pathogen and suggested to be responsible for some amphibian declines. It causes a disease (chytridiomycosis) that has since been linked to amphibian declines around the world [17]–[19]. Evidence of chytridiomycosis outbreaks at the times of population declines has led to the emergence of a strong paradigm: that Bd has recently spread across the globe, wreaking havoc on amphibian populations as it colonizes new sites [20].

Interestingly, the reported spatiotemporal patterns in amphibian declines are now the primary evidence cited for the spread of Bd (e.g., [15]) (although recent genetic data potentially also support the idea [21], [22]). Because of this, it is important to remember that an observed wave of population declines does not directly constitute evidence for the spread of a particular pathogen. Direct evidence for the spread of a pathogen comes from observation that the pathogen was absent at a site, but then appeared at some later date. Additionally, such evidence needs to come from multiple sites so that we can observe the spatial dynamics of pathogen invasion. For Bd, as with many other emergent diseases for which monitoring arose after population declines, the wave of pathogen invasion is only evidenced indirectly, via host declines (e.g., [14], [15]). In the case of Bd, strong evidence for a state-shift from absence to presence of Bd comes from only a handful of disconnected sites. At El Copé, Panama, for example, Lips and collaborators [18] sampled amphibians intensively for Bd in an initially healthy amphibian assemblage, but only detected it for the first time immediately before an epidemic outbreak of chytridiomycosis, which was followed by the collapse of the assemblage [15]. Their data indicate that Bd was likely absent at El Copé, then appeared and quickly reached high prevalences, which coincided with declines of many species. Because of the difficulty of obtaining such longitudinal data, however, this is probably the only locality in the tropics for which a clear link between the arrival of Bd and the decline of an amphibian fauna has been shown. A few sites in temperate regions may also have potentially useful longitudinal series (e.g., California, [19]). In isolation, however, these sites cannot demonstrate a spreading wave of Bd.

On Australia’s north-eastern seaboard, amphibian declines first occurred in southern Queensland in 1978, then in central Queensland in the mid 1980s, and commenced in northern Queensland in 1989 [14]. This was the first observation of the wave-like nature of declines and came several years before the discovery that Bd causes chytridiomycosis. The observation of Laurance and colleagues [14] was made without knowledge of Bd as a possible driver, although they speculated that some kind of “waterborne virus” might be to blame. Despite the fact that Bd was never mentioned in the paper, the Laurence *et al.* paper [14] is now commonly cited as evidence that Bd has spread in a wave-like manner (e.g., [2], [19], [23]–[25]). Given the massive research effort on Bd in the ensuing 16 years, and the fact that Australia has been a leader in this effort, is it now possible to conclusively demonstrate that this wave of declines was, in fact, driven by the invasion of Bd?

Such a demonstration is of more than historical interest. If, across multiple populations, there is little relationship between the dates of Bd arrival and population decline, then we are faced with the possibility that Bd is not the sole driver of declines. If Bd is even occasionally present, possibly at very low prevalence, for years before outbreaks, some other factor – such as the spread of an immunodeficiency virus, changes in climatic patterns [26]–[28]; or increased stress, possibly due to climate change [29] – must be involved in its emergence as a pathogen. Because of the severity and geographic extent of these declines, but also because amphibians have good historic records in museum collections and amphibian chytrid is detectable in museum specimens, it should be possible to test for the link between pathogen arrival and the timing of decline, and it should be possible to test this link in the place where the wave-like nature of declines was first noticed; north-eastern Australia. We attempt to do so here.

## Materials and Methods

The decline patterns presented in this paper stem from Laurance and collaborators [30] and the Bd data were recently published in an Australia-wide compilation of data from 1956 to 2007 [31]. To make our analysis consonant with the area discussed in Laurance [30], we restrict the Australia-wide data used to only that from the Australian east coast, north of Brisbane.

**Figure 1:**
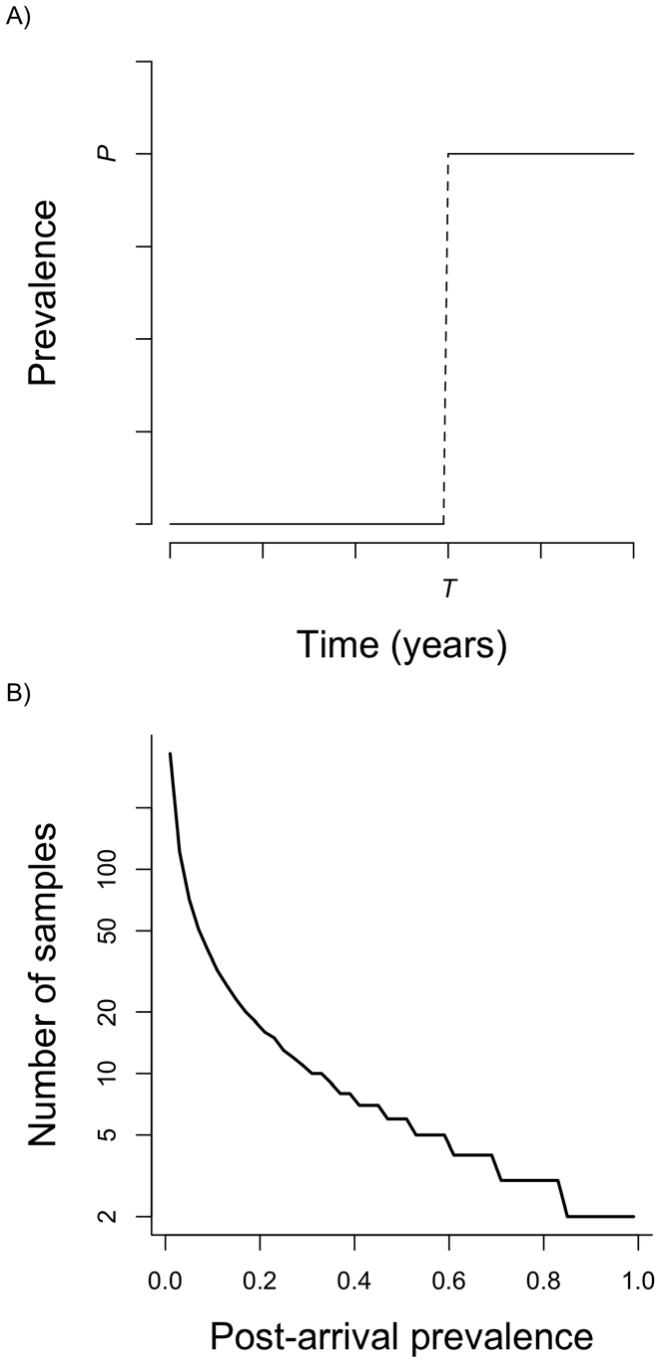
Model schematic, and the relationship between post-arrival prevalence and minimum adequate pre-arrival sample size. A) Schematic of the statistical model used to estimate the arrival time of chytrid. The model is a threshold where the prevalence of infection is zero up until some time, *T*, at which point the prevalence jumps to some new, average value, *P*. B) The approximate number of samples from a population with prevalence of zero required to provide a lower (2.5^th^ percentile) bound on the time of arrival (*T*) parameter in our model. The number of samples required scales with the post-arrival prevalence (*P*). This curve was generated by solving for the number of samples with zero positives required to reduce the binomial probability of that outcome below 0.025, given the stated prevalence.

Spatial data were collapsed into spatial bins for the purposes of estimating Bd arrival time at each locality. Bins were defined by the three localities (from Laurence *et al.* 1996) where declines are known to have occurred. We placed Bd sampling localities into the bin defined by the closest decline locality. This ensured that each Bd sampling locality was always associated with the closest decline locality (see Figure 1). For each of these spatially aggregated “populations”, we estimated the arrival time of Bd.

The basic premise of our analysis was to first estimate arrival date of Bd, and then regress the decline date against this estimated arrival date. A regression slope around 1 (with an intercept less than or equal to zero) would indicate a strong temporal link, across sites, between Bd arrival and population decline. This analysis would not only rigorously assess the link between arrival and decline across multiple sites, but could potentially give us important ecological information, such as the lag between Bd arrival and population decline (estimated in the intercept). Our plan was to estimate these regression coefficients in a hierarchical Bayesian framework, in which the uncertainty in our estimates of arrival time are incorporated into our final estimate of the regression coefficients. Unfortunately, the first step in this analysis (the estimation of arrival dates, described below) indicated that this hierarchical analysis would be pointless because there is almost no information in the data regarding Bd arrival date.

To estimate Bd arrival times at each aggregated locality, we used the simplest appropriate model possible: a threshold model, where prevalence is zero, up until a time (*T*), at which point prevalence moves to a non-zero mean (*P*; Fig. 1A). Although simple, the model points to the importance of possessing a substantial sample of individuals before the pathogen is first detected. This “pre-arrival” sample is critical for providing a lower bound on our estimate of *T*. The model also displays another important and unavoidable property: the lower bound on our confidence interval around *T* is strongly contingent on the value of post-arrival prevalence, *P* (Fig. 1B). If the post-arrival prevalence is high, then only a small sample size is required to be confident that Bd was not present before its first observation. Thus, sampling effort required to generate precision around arrival time will vary from site to site, but in all cases “pre-arrival” samples are needed to have any way of estimating the earliest possible date of pathogen arrival.

The threshold model was fitted for each site in a Bayesian framework using the JAGS Gibbs sampler [32], with uniform priors for arrival time, *T* (Uniform between 1950–2010) and *P* (Beta (1, 1), which is uniform between zero and one). Restricting the priors for *T* to between 1950–2010 was done for logistical reasons rather than any belief that Bd did not exist before 1950. Thus, 95% credible interval bounds that approach either 1950 or 2010 should be treated as conveying no information on actual arrival time at that bound. The ability of the model to estimate parameters was confirmed using simulated data in which prevalence values followed the threshold model exactly, but “observed” data were drawn from this expectation using random draws from a binomial distribution. When sample sizes in simulated data before and after *T* were sufficient, the model successfully recovered parameter values across a range of *T* and *P*.

Posterior densities for *T* and *P* were estimated from three chains each with 100,000 samples following a burn-in of 10,000 iterations. In both simulated and real data sets, convergence across chains was confirmed using Gelman and Rubin’s ‘potential scale reduction factor’ (which almost always gave point estimates of one for all parameters, confirming convergence between chains). All data manipulation and analysis was conducted in R [33], and the scripts and data are available from BLP upon request.

## Results

**Figure 2:**
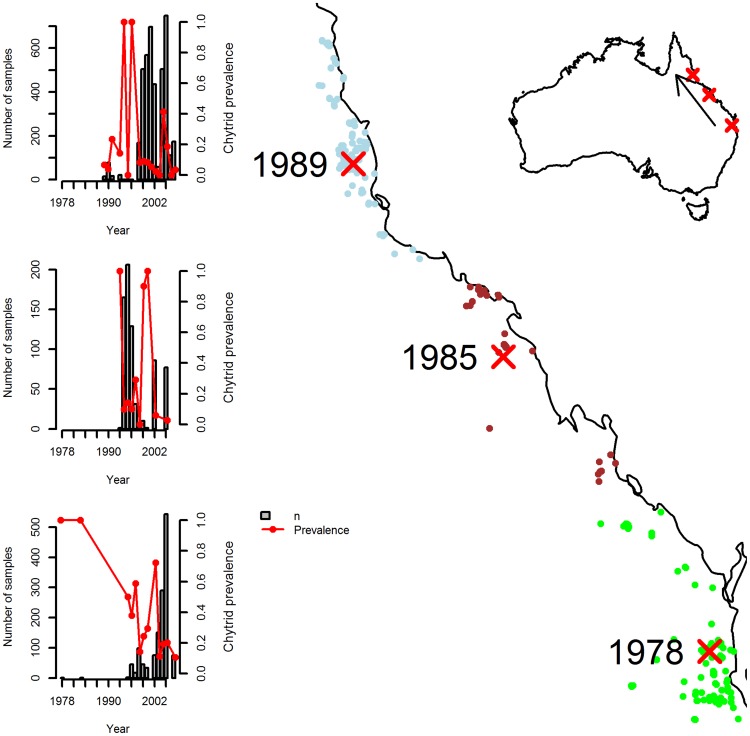
Distribution of localities sampled for Bd along the north-east coast of Queensland, Australia. Decline localities are marked with a large red cross and labelled by their approximate date of amphibian population decline. Left-hand-side inset panels show sample sizes (grey bars) and prevalence (red series) by year for data aggregated around each of the decline localities. Note that earliest samples indicate the presence of Bd, and that there are no earlier samples indicating its absence.

**Figure 3:**
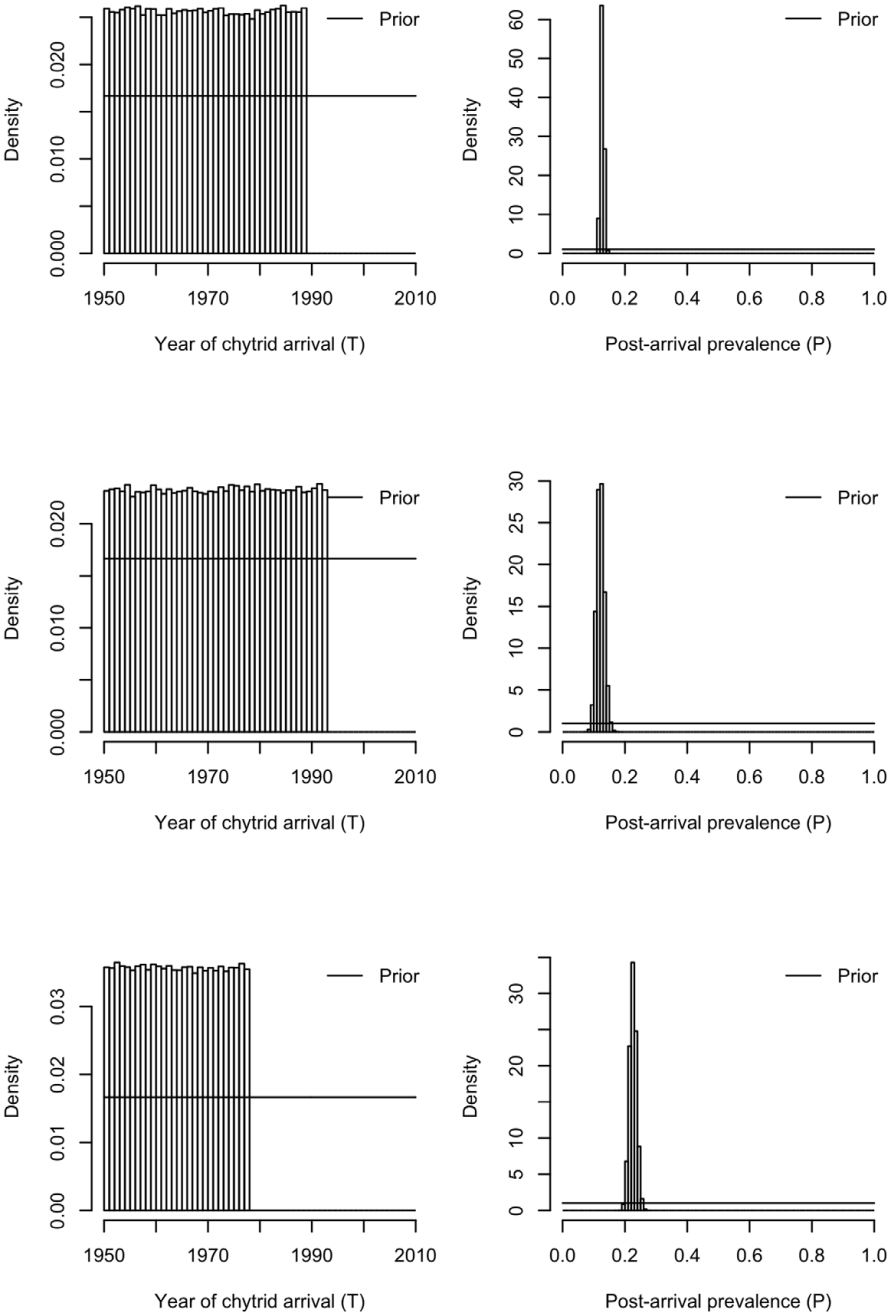
Posterior parameter estimates for arrival time (*T*, left hand column) and post-arrival prevalence (*P*, right hand column) of *Batrachochytrium dendrobatidis* at the three binned localities. Each row in the panel represents the parameter posteriors for each site (where sites are arranged from North to South as per Fig. 2). Figures report histograms of 300,000 samples (across three chains) from the posterior distribution of each parameter. Solid lines show the prior distributions in each case. Posteriors for arrival time show lower bounds that are indistinguishable from those of the priors, indicating no information in the data with which to estimate these lower bounds. Post-arrival prevalence (*P*), on the other hand, is tightly estimated at each site.

Figure 2 shows the spatial binning of the dataset and the spread of sample effort and prevalence over time at each binned locality. Importantly, at all three binned localities there are no “pre-arrival” samples: there are no data on the prevalence of *Batrachochytrium dendrobatidis* (Bd) before it was first detected (Fig. 2). Thus, at all three localities we have no information confirming the absence of Bd prior to the date of first sampling. Figure 3 shows the resulting estimates of Bd arrival time from the threshold model. As expected from the dearth of “pre-arrival” information, the lower bound of arrival time is identical to the lower bound of our prior. Thus, in north-eastern Australia, the model confirms that it remains possible that Bd was present (from months to thousands of years) before it was first observed.

## Discussion

Surprisingly, our analysis suggests that in north-eastern Australia – across the sites where the wave-like pattern of amphibian decline was first observed and where intensive research has been conducted ever since – there is no evidence that the arrival of *Batrachochytrium dendrobatidis* (Bd) coincided with amphibian population decline. The reason for this is that we effectively have no idea when Bd arrived at each of the decline localities. If we have no idea when it arrived, we cannot claim to know that it arrived immediately before the populations declined. Our analysis is not evidence against the role of Bd in amphibian declines, or against its recent range expansion, but clearly points to a missing link in the logical argument linking the spatiotemporal pattern of amphibian decline to the spread of Bd.

The threshold model presented in this manuscript is the most basic analysis that should be performed on data when seeking an arrival time. However, in all binned localities, Bd is present in the first sampling period. Thus, there are no sampling periods before the first detection of Bd and therefore no information on the earliest possible arrival date of Bd at each binned locality. There is no information in the data and so, to some extent, the choice of model is irrelevant. Nonetheless, we have developed this model as it is a very useful tool for understanding why the missing data matters, and serves as a useful demonstration of the need for those data. Without them, our lower bound on arrival time will remain unresolved.

It is clearly important for conservation efforts to proceed, however, and one of the factors that must be understood and controlled is the spread of Bd. It is, nonetheless, critical to test the assumption that the presence of Bd is the only important factor. In the absence of tests it remains possible that the emergence of epidemic chytridiomycosis is caused by factors other than the spread of the pathogen, and even that Bd has been present for an extended period in amphibian populations in north-eastern Australia, and that some other factor triggered the onset of epidemics and amphibian population declines.

Clearly, to resolve this issue in Australia we need to better target our data collection. In particular, we need to increase the amount of data on the prevalence or presence of chytrid *before* declines were observed. The reason that the Australian data lack information on arrival time is because there are no data on Bd prevalence from before the dates of population declines (Fig. 2). This is not from lack of specimens (museums in Australia have collections of thousands of specimens dating back to the 1800s), but rather from lack of analysis of those specimens. Detection of Bd in museum specimens has traditionally been hampered by our inability to extract and amplify DNA from formalin-fixed museum specimens. Happily enough, however, this limitation has recently been removed: new techniques are now available with which to detect Bd in old museum specimens [13], [34]. Combining these techniques with careful double-checks using histological examination will yield the data that we need, and is critical if we are to advance our understanding of Australian (and, indeed, the global) amphibian declines.

Our analytical framework suggests that the most effective way to increase precision of estimates of the date of arrival of Bd will be to start screening specimens from the earliest date of confirmed Bd presence in an area, working backwards in time (Fig. 1). In this way, we can concentrate our sampling density on the time when it matters most: immediately before the putative arrival date. Dense sampling in this period, if it yields zero prevalence, will rapidly compress our confidence intervals around arrival date for Bd, and allow us to make much clearer inferences regarding the correlation between Bd arrival and population decline. We also suggest that, to be conservative, sampling should aim to establish an upper 95% confidence limit for pre-arrival prevalence that is below 5% before concluding that Bd is absent. This requires a sample of 72 or more individuals with zero prevalence (Fig. 1B).

Although several waves of amphibian declines have been observed throughout the world (e.g., [15], [19]), none of them have made a quantitative assessment of the link between the arrival date of Bd and the decline date of multiple amphibian populations; indeed ours is the first attempt to rigorously estimate arrival dates of Bd in a region anywhere in the world. Nonetheless, sufficient data likely exist to quantify arrival dates for at least some localities (e.g., [13], [19]) and such quantification should be done. It is, for example, already clear that substantial lags between introduction of the pathogen and the decline of the population can occur (e.g., Figure 2B, [19]). How long were these lags, and what caused the system to transition to one of population decline? Was it density dependent population dynamics [35]; climate shift [27], [29]; extreme weather events; or the invasion of novel Bd genotypes [21] Without clear bounds on the potential arrival date of Bd, elucidating the factors that interact with Bd to drive population collapse will continue to be fraught with uncertainty [36]. Quantification of Bd arrival dates is, thus, a clear priority, and applying the new Bd detection technologies [13], [34] in a systematic way to museum specimens is clearly an important direction in research on Bd and the amphibian declines in general.

More generally, our discovery of a complete lack of evidence linking the arrival of Bd and the timing of population declines in north-eastern Australia points to the alarming ease with which paradigms can become established even in the absence of critical tests of those paradigms. The Laurence et al (1996) paper is commonly cited as evidence for the recent spread of Bd, but that paper makes no mention of Bd, and data collected on Bd prevalence since then (analysed here) cannot be used to link Bd arrival and frog population declines. That this should occur in one of the world’s most intensively studied regions for chytrid ecology and dynamics, and in a place where the spatiotemporal pattern of amphibian declines was first observed, is sobering. Given the difficulty of detecting and monitoring emergent diseases of wildlife, however, it may often be the case that host decline dates are used as a proxy for pathogen arrival date (e.g., [4], [37]). While this is a useful first step, we should be aware that we are using a proxy, and should be clear that doing so makes it difficult to rule out other potentially important drivers of population decline. A rapid transition to a system that monitors the pathogen and, ideally, reconstructs the historical dynamics of that pathogen is critical if we are to advance our understanding of emerging wildlife diseases.
